# Multi-Objective Path Optimization in Fog Architectures Using the Particle Swarm Optimization Approach

**DOI:** 10.3390/s23063110

**Published:** 2023-03-14

**Authors:** Nerijus Morkevičius, Agnius Liutkevičius, Algimantas Venčkauskas

**Affiliations:** Department of Computer Science, Kaunas University of Technology, 44249 Kaunas, Lithuania

**Keywords:** fog computing, internet of things, path optimization, multi-objective optimization, particle swarm optimization, analytic hierarchy process

## Abstract

IoT systems can successfully employ wireless sensor networks (WSNs) for data gathering and fog/edge computing for processing collected data and providing services. The proximity of edge devices to sensors improves latency, whereas cloud assets provide higher computational power when needed. Fog networks include various heterogeneous fog nodes and end-devices, some of which are mobile, such as vehicles, smartwatches, and cell phones, while others are static, such as traffic cameras. Therefore, some nodes in the fog network can be randomly organized, forming a self-organizing ad hoc structure. Moreover, fog nodes can have different resource constraints, such as energy, security, computational power, and latency. Therefore, two major problems arise in fog networks: ensuring optimal service (application) placement and determining the optimal path between the user end-device and the fog node that provides the services. Both problems require a simple and lightweight method that can rapidly identify a good solution using the constrained resources available in the fog nodes. In this paper, a novel two-stage multi-objective path optimization method is proposed that optimizes the data routing path between the end-device and fog node(s). A particle swarm optimization (PSO) method is used to determine the Pareto Frontier of alternative data paths, and then the analytical hierarchy process (AHP) is used to choose the best path alternative according to the application-specific preference matrix. The results show that the proposed method works with a wide range of objective functions that can be easily expanded. Moreover, the proposed method provides a whole set of alternative solutions and evaluates each of them, allowing us to choose the second- or third-best alternative if the first one is not suitable for some reason.

## 1. Introduction

In recent years, the Internet of Things (IoT) has expanded significantly, leading to a large amount of data being generated by IoT devices. These data are sent over various networks to cloud-based servers and other data consumers. To cope with this large amount of data, decentralized fog-based architectures can be used. This allows ensuring low latency and effective resource usage since the IoT data can be processed close to the data sources. Fog networks consist of heterogeneous fog nodes and edge devices with different resource constraints, such as battery level, security level, central processing unit (CPU) use, and memory use. Moreover, fog nodes as well as user end-devices can be mobile (e.g., vehicles, smartwatches, smartphones, and mobile sensors), which means that the fog network architecture is not static, but dynamic, with a self-organizing ad hoc structure. This leads to the two major problems related to service provisioning in fog networks: optimal service (application) placement in the fog node and optimal data routing between the user end-device and the fog node that provides those services.

Each time the user end-device asks the nearest fog node to provide some services for it, the fog-based system should decide on the best fog nodes for service placement, considering various constraints when searching for the optimal placement, including battery level, CPU use, memory use, and security level. Recently, a lot of research effort has gone into developing optimal application and service placement algorithms and architectures for fog networks, which are reviewed in [1,2,3,4]. However, usually, optimal placement is solved as a separate problem and the cases when services are placed in fog nodes not adjacent to the user end-device are ignored. In such cases, fog systems need to route the IoT data from the user end-device to the fog nodes where required services are placed. These data are routed along a path starting at the user end-device (data source) passing through several fog and edge nodes until the data reach the final node or nodes, which process the data. Fog systems consist of heterogeneous nodes with different computational capacities and constraints, and the routing algorithm should consider these constraints while calculating the best path to route the data. For example, the path should exclude nodes with insufficient computational or energy resources or nodes that have low security levels if the user application requires a high level of data protection. Moreover, the algorithm for determining the data path should be simple and lightweight enough to be able to run on constrained devices, such as fog nodes, and provide a good enough path for data transfer. In such a scenario, it is not important to locate the optimal path as long as the path found using a lightweight algorithm addresses all the requirements and ensures good overall results. In such applications, heuristic optimization methods are usually used.

Therefore, data routing in heterogeneous fog networks becomes a multi-objective optimization task, which considers various computational, resource, and security constraints of the fog and edge nodes, and identifies the best path in terms of optimizing latency, energy consumption, bandwidth, etc. There are usually several alternative routing paths, which should be evaluated in real time to achieve the lowest possible response time and latency. Since fog network nodes (especially edge nodes) usually have limited resources, they rapidly reach their capacity, leading to longer and more complex paths from the user end-device to the remote fog node(s) suitable for service(s) placement. Moreover, the mobility of user end-devices and even fog nodes adds another level of complexity, requiring the algorithm to perform constant service replacement and, thus, constantly recalculate the best routing path.

Over the years, various researchers have solved the optimal routing or path-finding problem. Most of these works in the field of wireless sensor networks (WSNs), fog, and IoT systems focus on optimal routing protocols and data forwarding techniques, which in most cases have a single objective, e.g., optimize energy usage [5,6,7]. As concluded in [5], one of the drawbacks of existing routing techniques is fixed static routing and the reliability of decision-making nodes. The survey dedicated solely to nature-inspired algorithms for WSNs [8] concluded that these algorithms are well-suited for solving multi-objective real-world optimization problems, while traditional algorithms fail to provide satisfactory results because the problem is complex. Nature-inspired algorithms, including particle swarm optimization (PSO), are used for energy-efficient clustering and routing, optimal coverage, data aggregation, and sensor localization, as classified in [8]. The systematic study of topology control methods and routing techniques in wireless sensor networks [7] reviewed recent articles, including topology-aware PSO and ant colony optimization (ACO)-based routing techniques in static and mobile wireless sensor networks, and stated that these techniques do not incorporate delay-sensitive routing or timely data delivery. According to [7], the latest routing algorithms based on PSO and ACO techniques lag where the topology and routing requirement are delay-sensitive and concerned with data delivery ratio, throughput, and quality of service (QoS). Although several real-time routing techniques attempt to satisfy minimum latency and maximum throughput, the study [7] concluded that the research in this field is in an early stage and limited to some well-known protocols. These findings are further supported by swarm-intelligence-based optimization techniques for the WSN survey [9], which concluded that there are still some open research challenges, including weighing the energy consumption, QoS, security, and reliability of the network. In the survey in [9] also, the authors found that most of the previous work optimized the performance of WSNs only from a single perspective (a single objective).

In [10], the authors reviewed the multi-objective optimization techniques and challenges in WSNs, stating that in WSNs, routing is an essential factor that should be performed optimally, though there are various routing challenges, including scalability, energy consumption, connectivity, deployment, security, and coverage. According to [10], multi-objective routing algorithms should consider coverage, throughput, end-to-end delay, capacity, collision, etc. Existing routing algorithms usually avoid security, though it is an essential factor in WSNs [10]. In [11], the authors reviewed path optimization techniques and noted that most path optimization techniques use performance measures, including packet delivery rate, network lifetime, energy consumption, delay, and distance, but do not consider the analysis of messages and the time complexity of their techniques. Therefore, real-world implementations of the proposed techniques pose a significant challenge as well [11]. Finally, the survey on path planning for mobile sink in IoT-enabled WSNs [12] paid attention to the fact that most research assumes an obstacle-free network environment, while in a real environment, obstacles are usually present.

To counter the aforementioned problems, we propose a multi-objective PSO-based path optimization method that can identify the best routing path from the user end-node to the fog node(s) where services for the user device are placed. Our method not only considers multiple constraints of the fog nodes along the path (CPU, memory, battery, security, etc.), but also supports the mobility of the user and fog nodes, as well as failures of the nodes, constantly reconfiguring the fog network and path. Instead of routing the IoT data dynamically, until it reaches the suitable fog node for further processing (which does not guarantee the optimal path), our method calculates the optimal path each time a change occurs in the fog network, such as user end-node movement, computational resource changes in the fog nodes, and fog node failures. This algorithm selects the best of all possible paths and forwards the data directly to the processing fog node(s). As a result, latency and response time are low and bandwidth and energy usage are minimized, achieved by combining the distributed orchestrator model, proposed by us in [13], and the PSO-based optimization method, which locates the best path from the user end-node to the fog node(s), where the distributed orchestrator places the services for that user device. The distributed orchestrator constantly synchronizes the computational resources and constraints of each fog node, which allows the PSO-based algorithm to straightaway identify the optimal (suboptimal) path, instead of using various dynamic or evolutionary approaches, which achieve good results only after some time. Therefore, we propose a method that is able to simultaneously cope with two major problems in fog networks: optimal service (application) placement and optimal routing.

Heuristic nature-inspired algorithms, such as PSO [14], genetic algorithm (GA) [15], and ACO [16], or even the cuckoo search algorithm (CS) [17], help identify the optimal path in similar applications. The objective functions used in these methods vary from maximization of the packet delivery rate to data transmission latency, overall power consumption, delay time, and minimization of bandwidth consumption. Most methods tend to identify an optimal path for data flow based on one, most important, parameter of the IoT system or by combining several characteristics, such as latency, bandwidth, or energy, into one composite criterion using a simple linear combining objective function. However, using a composite of many criteria is not always the ideal solution to this issue because it is challenging to correctly determine the weights of the individual criterion. The authors of [18] recommend the use of simulation and trial and error to adjust the weights of the criteria for constructing the linear combined function. One of the possible solutions to this problem is to use multi-objective optimization to identify all nondominated data paths, that is, the Pareto Frontier of the problem solutions [19]. The final challenge in such cases is to choose the best solution from all nondominated candidates. Several different approaches may be used to compare the alternatives. If the application area is fixed, well defined, and extensively investigated in advance, some form of aggregation function of several competing factors can be constructed using mathematically calculated decision matrices [20]. As the final step in the optimization process for selecting the best path from all alternatives, we propose using the analytic hierarchy process (AHP) [21]. AHP uses only simple pairwise comparisons of all alternatives using all objective functions and can be easily adapted for use by machine-based decision making [22,23,24]. The values of all criteria are normalized, allowing us to use heterogeneous measurement scales for different objective functions. The importance of the criteria used to construct the decision matrix is also evaluated using the same methodology, allowing us to skip the most controversial step, that is, manual weight assignment to different criteria. The decision matrix is prepared in advance by the experts in the application area and used during the execution of the algorithm. Being deterministic and easy to implement, the AHP fits the constrained nature of the fog nodes well.

Our main contribution to the field of data path optimization in fog architectures is a novel two-stage optimal-path-finding algorithm based on the multi-objective particle swarm optimization and the analytical hierarchy process. During the first stage of the proposed method, a Pareto set of nondominated alternative paths is found. Then, AHP is used to choose the best path according to the provided application-specific judgment matrix.

The article is organized as follows: Section 2 presents the conceptual model of the fog system and a formal definition of the best data routing path finding problem. Section 3 presents the proposed two-stage multi-objective optimization method to determine the best data routing path. Section 4 covers the experimental evaluation and discusses the results obtained. Section 5 concludes the article.

## 2. Problem Definition

### 2.1. Conceptual Fog System Model

In this section, we present the fog architecture model that was used to formulate the path optimization problem and define its properties and constraints. Modern fog architectures are multilayered systems usually based on the fog computing model defined by the OpenFog Reference Architecture and adopted in the IEEE 1934-2018 standard [25]. Such architectures have three layers: the IoT device layer (end layer), the layer of fog nodes (fog layer), and the cloud layer (containing at least one cloud data center):

- The bottom layer of IoT devices is widely distributed geographically and closest to end-users. It contains various user end-devices, such as cameras, mobile phones, smart cars, and smartwatches. The IoT data and various user service requests generated in this layer are forwarded to the middle fog layer for further processing and storage.
- The middle fog layer contains heterogeneous devices that are able to process user requests and provide services for the users. These devices include various access points, routers, gateways, switches, base stations, fog servers, etc. They are connected to the cloud servers and can offload computationally demanding tasks to the cloud. Fog nodes can be static (e.g., traffic cameras) or mobile (e.g., vehicles and drones).
- The upper cloud layer usually consists of several servers and is used for resource-intensive computations and the storage of large volumes of data.

**Figure 1:**
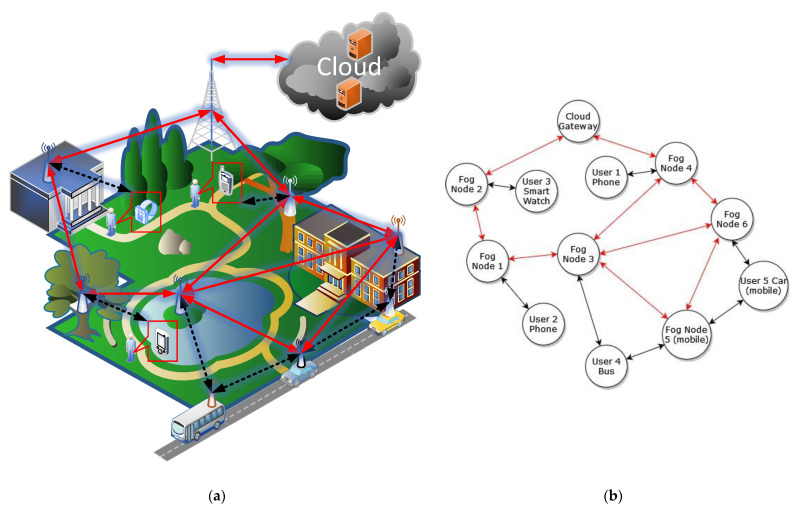
A heterogeneous ad hoc fog network architecture with mobile user devices and static and mobile fog nodes: (**a**) a conceptual model of the fog architecture of the smart campus of a university. The arrows represent the data routing paths between the fog nodes, user end-devices, and cloud servers. (**b**) A graph representation of the fog architecture. The graph nodes represent the fog nodes and user devices, and the graph edges represent the communication paths.

Figure 1a shows a sample fog architecture of the smart campus of a university. It is made up of heterogeneous mobile user end-devices (smartphones, smartwatches, smart cars, etc.), various static and mobile fog nodes (access points, routers, gateways, moving vehicles, etc.), and cloud servers to which fog nodes can send data. The solid red arrows in Figure 1a represent the data paths between the fog nodes, while the black dotted arrows show the connections between the user end-devices and the fog network. The user devices (the smartphones, the smartwatch, the bus, and the yellow car), as well as some fog nodes (a blue car), are constantly moving. Therefore, the user end-devices and fog nodes form a dynamically changing ad hoc network with frequent reconnections and service relocation, requiring the calculation of new routing paths. Each time the user device requests services from the nearest fog node, or the user device moves between fog nodes, or a fog node fails to provide services, the requested services are moved (placed) to the most suitable fog nodes to meet the user application requirements and optimize system performance. Since it is not necessary that the services be placed in the adjacent fog nodes, the best path should be found from the user end-device to the fog node(s) where the services were placed.

Each fog node (shown as a wireless antenna in Figure 1a) has constantly changing computational resources, such as the CPU use level, the memory use level, the remaining battery level, and the security level. These parameters are used as optimization constraints for both placing services and finding the best route. Furthermore, each communication path segment (shown as an arrow in Figure 1a) has its own constraints, such as bandwidth, latency, and security. These constraints should also be considered when finding the best route. Usually, there are several alternative routes, one of which should be chosen according to the selected optimization objectives, for example, overall network energy consumption, latency, and bandwidth usage.

Therefore, finding the best route in heterogeneous fog networks, considering various fog node and communication channel constraints, becomes the multi-objective optimization task. The most popular way to describe this problem is the application of graph theory, where fog nodes and end-devices become the graph nodes, while communication connections become the directed edges of the graph, as shown in Figure 1b. Depending on its computational and communication constraints, each node and edge of the graph adds some cost (weight) to the transfer of the data. Therefore, the best path to route data is one with the smallest overall cost and may not be the shortest one.

### 2.2. Application of PSO to Identify an Optimal Path

The particle swarm optimization (PSO) algorithm was originally intended for optimizing continuous problems. Some modifications [26,27] allow it to be used to solve discrete problems, including both discrete objective function and discrete area of definition. In the case of multi-objective optimization, the PSO algorithm may be adapted to determine a Pareto optimal set of solutions [28,29].

The main challenge of using PSO (as well as GA) as the algorithm to identify the optimal route is encoding the route (or path in the graph) into a particle (or chromosome in the case of GA). Four required properties of such mappings are discussed in [15]. Such encoding is not trivial and affects the overall performance of the whole path search process. After evaluating the different encoding schemes [30,31], two classes of encoding approaches can be distinguished: direct representation of the particle path and indirect representation of the particle path.

If direct representation is used, then the particle in PSO is composed of a sequence of node IDs that form the path that starts at a source node and ends at the destination node [15,32]. The main disadvantage of this approach is that the particles are of different lengths as the paths in the graph may include different numbers of nodes. Moreover, a random sequence of node IDs may not correspond to a valid path, may not terminate at the destination node, or may form a loop, considerably increasing the number of invalid particles. In this case, a discrete version of PSO should be used, which causes problems in forming the area of definition because the particles are of different dimensions.

An indirect encoding scheme was proposed by Gen et al. [33]. In this case, the particle encodes some kind of “guiding information” about the nodes that corresponds to the path. This guiding information can be about the priority in which the network nodes should be included in the path. The particle is initialized by assigning random priorities. Then, the path is generated by sequentially appending the nodes starting from the beginning node. During each step, the subsequent node with highest priority is chosen from all possible nodes according to the graph. The process stops when the destination node is reached. This approach has some advantages over direct encoding schemes. All particles have the same dimensions, and these dimensions are equal to the number of nodes in the network. The continuous space version of PSO may be used, which is more responsible for fine-tuning the optimization parameters. Compared with direct encoding, the probability of valid path generation is greatly increased, causing fewer (but not fully eliminating) invalid particles that form paths with loops or do not reach the final node.

### 2.3. Formalization of the Path Finding Problem

**Figure 2:**
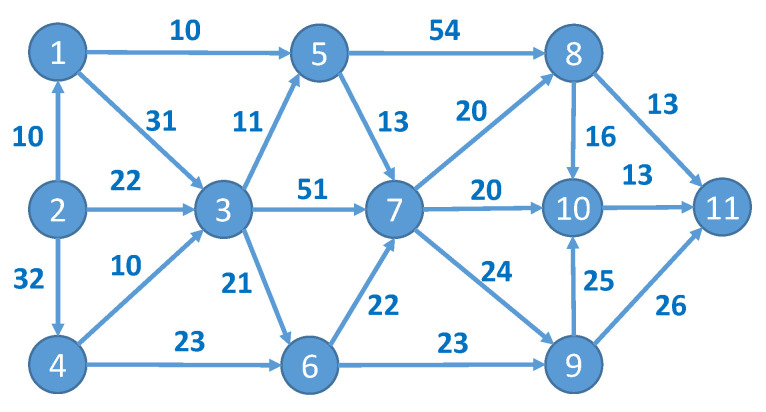
A sample graph representing the fog network.

The main objective is to determine an optimal data path from the sensor to the data processing node (the service) on the basis of multiple criteria, such as the lowest possible latency, energy consumption, random-access memory (RAM), and CPU use. We describe this path in terms of graph theory and the shortest path problem. The entire IoT infrastructure is represented as a directed graph G=(V,E), which comprises a set of nodes $V=\left\{v_{i}\right\}$ and a set of edges E∈V×V connecting nodes $v_{i}$, i=1, 2, …, n. The non-negative number $w_{ij}$ is assigned to each edge-connecting node $v_{i}$ and $v_{j}$ and represents the cost of data transfer between these nodes expressed in units (e.g., ms for latency and percent or kbps for bandwidth use) corresponding to the objective function used for optimization. The first node ($v_{1}$) is the data source and the last node ($v_{n}$) is the data destination node. A valid path π for data transfer is a sequence of edges $\left(v_{1},\;v_{x}\right)$, $\left(v_{x},\;v_{y}\right)$, …, $\left(v_{z},\;v_{n}\right)$ from set E in which no node appears more than once. For simplicity, this path can be equivalently expressed as an enumeration of nodes $\left(v_{1},v_{x},\;v_{y},\ldots ,v_{n}\right)$. For example, the valid path for the graph presented in Figure 2 may be expressed as $\left(v_{1},\;v_{3}\right)$, $\left(v_{3},\;v_{7}\right)$, $\left(v_{7},\;v_{10}\right)$, $\left(v_{10},\;v_{11}\right)$ or, in brief, as $\pi =\left(v_{1},v_{3},\;v_{7},v_{10},v_{11}\right)$. The total cost of this path is 31+51+20+13=115.

**Figure 3:**
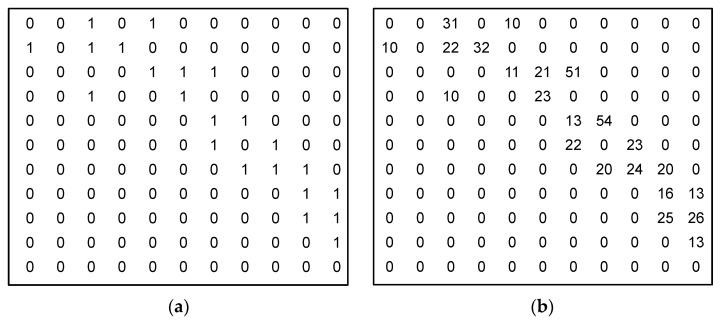
The matrices corresponding to the graph presented in Figure 2: (**a**) edge matrix $W^{\prime }$ showing the edges of the graph and (**b**) edge weight matrix W showing the edge weights.

For practical reasons, the costs of the edges are represented by the n×n matrix W, where elements $w_{ij}\in W$, i, j=1, 2, …, n represent the cost (or weight) of the edge $\left(v_{i},\;v_{j}\right)$. Figure 3b presents an example of an edge matrix, which shows the weights of the edges for the graph in Figure 2. If the upload and download capabilities of some nodes are asymmetrical, then this matrix is nonsymmetrical ($w_{ij}\neq w_{ji}$). Strictly speaking, in some cases, a situation where the edge $\left(v_{i},\;v_{j}\right)$ exists but $\left(v_{j},\;v_{i}\right)$ is absent, i.e., some client–server IoT protocol is used between the nodes, is also possible and all data transfers should be initiated only from the client side (e.g., CoAP protocol). For practical reasons, matrix W is complemented by edge matrix $W^{\prime }$ (Figure 3a), $w_{ij}^{\prime }\in W^{\prime }$, i, j=1, 2, …, n, where $w_{ij}^{\prime }=\{\begin{array}{c} 1,\;\mathrm{if}\;\mathrm{edge}\;\left(i,\;j\right)\;\mathrm{exists}\\ 0,\;\mathrm{otherwise}\; \end{array}$.

The objective was to find an optimal data path from node $v_{1}$ to node $v_{n}$ according to multiple criteria. We needed a multi-objective optimization method suitable for determining the shortest path according to m objective functions $f_{j}\left(\pi \right)$, j=1,2,…,m and the given constraints and conditions. The QoS parameters of the i-th possible data transfer path $\pi_{i}$ are expressed by the values of the objective functions $f_{j}\left(\pi_{i}\right)$, j=1,2,…,m. The result of function $F\left(\pi_{i}\right)=\left(f_{1}\left(\pi_{i}\right),f_{2}\left(\pi_{i}\right),\;\ldots ,f_{m}\left(\pi_{i}\right)\right)$ is the vector representing the length of the path $\pi_{i}$ according to all objective functions. All constraints are given by the following equation:

(1) $$\{\begin{array}{c} g_{k}\left(\pi_{i}\right)\geq 0,\;k=1,\;2,\ldots ,n_{g}\\ h_{l}\left(\pi_{i}\right)=0,\;l=1,\;2,\ldots ,n_{h} \end{array}.$$

Then, the main objective of the optimization process is to find the optimal path $\pi_{opt}$ that minimizes all objective functions $f_{j}$:

(2) $$\pi_{opt}=arg\underset{i}{min}\;F\left(\pi_{i}\right).$$

The objective functions may include the overall security of the whole system $f_{sec}\left(\pi \right)$, CPU usage $f_{CPU}\left(\pi \right)$, RAM usage $f_{RAM}\left(\pi \right)$, network bandwidth usage $f_{bw}\left(\pi \right)$, ), network latency $f_{lt}\left(\pi \right)$, power usage $f_{pw}\left(\pi \right)$, energy usage $f_{en}\left(\pi \right)$, etc. Table 1 summarizes all the key notations used in this paper.

## 3. Two-Stage Multi-Objective Optimization Method for Finding the Best Data Path

**Figure 4:**
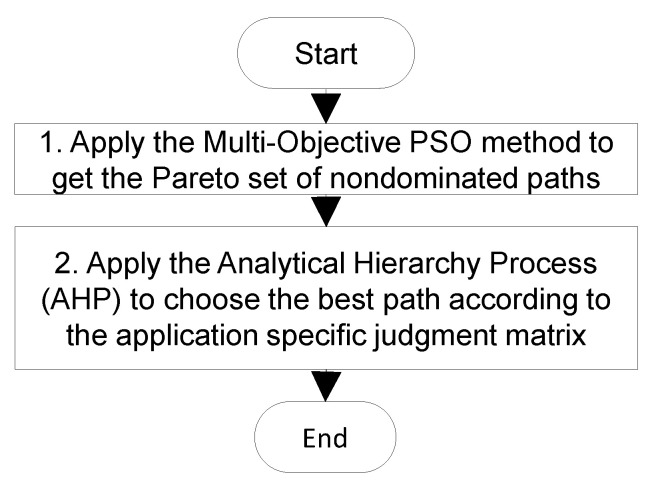
A flowchart of the proposed method for finding the best data path.

In real applications, the objective functions $f_{j}\left(\pi \right)$, j=1, 2, …,m contradict each other. For example, the highest security increases CPU and RAM usage. One of the obvious approaches used in many solutions is to combine all objective functions into one composite criterion using simple linear equations. In this case, it is difficult to choose the “proper” coefficients, especially when the number of criteria increases. We propose the use of the two-stage optimization process presented in Figure 4.

In step 1, the multi-objective particle swarm optimization (MOPSO) method was used to determine a Pareto set of nondominated solutions to the problem. In step 2, the analytical hierarchy process (AHP) [21,34] was used to choose the best solution from the Pareto set. The AHP uses the application-specific judgement matrix that represents the importance of objective functions in the specific application area. These matrices may be constructed beforehand by experts in the field using a simple pairwise comparison of criteria.

### 3.1. Multi-Objective Particle Swarm Optimization for Finding a Pareto Set of Alternative Paths

**Figure 5:**
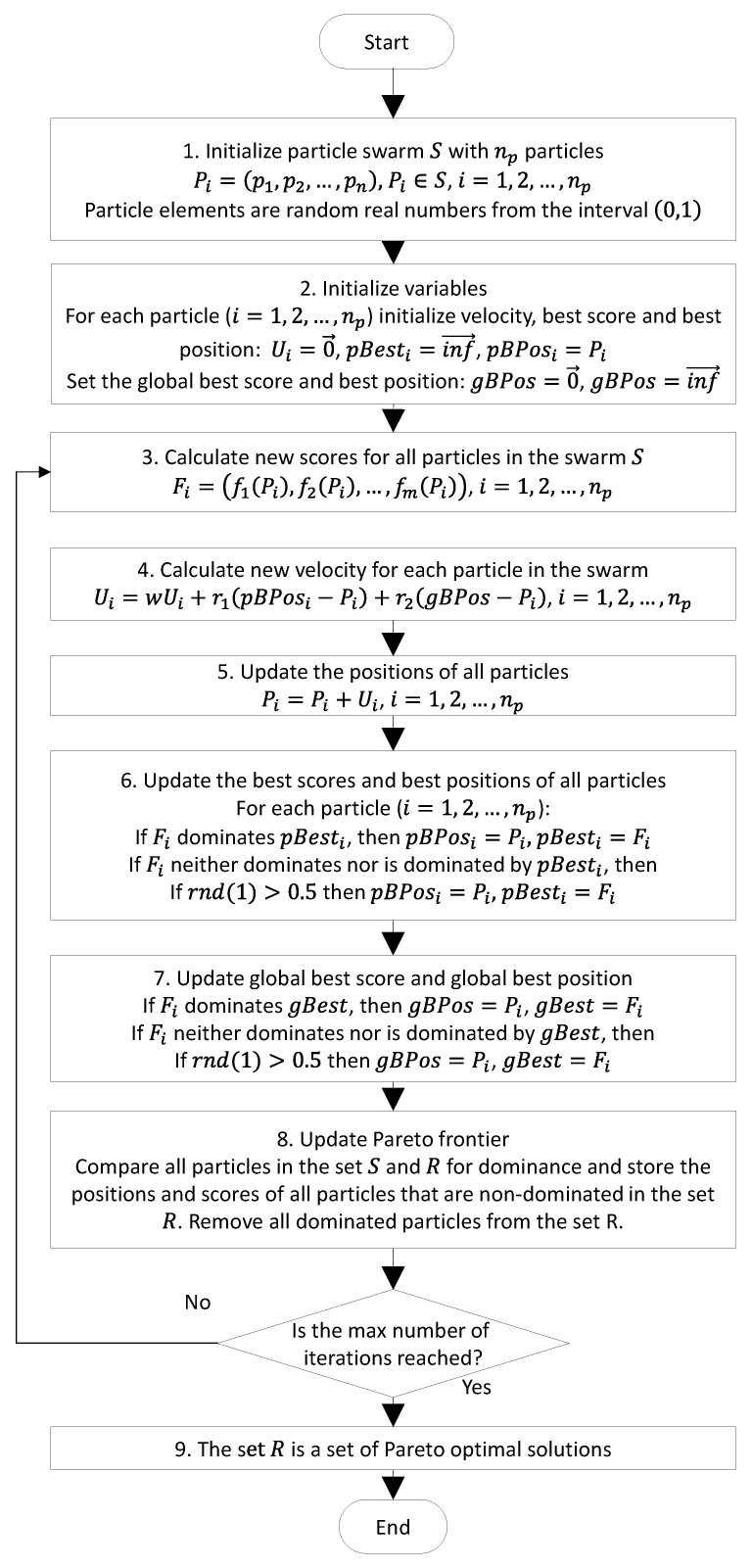
A flowchart of the multi-objective particle swarm optimization process for finding the set of Pareto optimal paths.

The PSO is inspired by the behavior of flocking birds. Individuals in the swarm are called particles and have assigned velocities. The particles fly through the search space according to personal experience and are also attracted by the best individual of the swarm. The MOPSO method proposed by Coello et al. in [29] was used to find the optimal path. This modification of continuous-space PSO tries to find a Pareto optimal (also called a Pareto Frontier) set of solutions. The Pareto set includes all nondominated solutions, meaning that each solution in this set is better than all other solutions according to at least one optimization criterion. Figure 5 presents a generalized flowchart of the multi-objective particle swarm optimization process used to find the Pareto set of paths.

In step 4, w was an inertia weigh parameter of the PSO algorithm. Initially, its value was 0.4. The coefficients $r_{1}$ and $r_{2}$ are random numbers in the range of [0, 1]; $U_{i}$ is the velocity of the i-th particle.

In step 5, the new position of the particle was calculated. If the particle was outside the definition range (i.e., one of the elements of the particle had a negative value), it was given an opposite direction of the speed ($U_{i}=-U_{i}$) and the position of the particle $P_{i}$ was set to the edge of the range of its definition (i.e., the search space).

In steps 6 and 7, the function rnd(1) generated a uniformly distributed random number from the interval [0, 1].

For particle encoding, we used the indirect (sometimes called priority-based) encoding approach. Each particle P, $P=\left(p_{1},p_{2},\ldots ,p_{n}\right)$, $p_{j}\in R$, $p_{j}\geq 0$, j=1, 2,…,n, represents one possible path in the graph from the first node to the last (destination) one. The elements of the particle are the probabilities of the corresponding nodes used during the construction of the path from the particle. When the new particle was generated, the elements of the particle vector were populated with random real numbers from the interval (0,1). Algorithm 1 describes the construction of the path corresponding to the particle:
**Algorithm 1:** Path construction algorithm**Input parameters:** graph G=(V,E) defined using edge matrix $W^{\prime }$, particle $P=\left(p_{1},p_{2},\ldots ,p_{n}\right)$.
Include the first node in path π: $\pi =\left(v_{1}\right)$, curr=1.$\mathrm{Initialize}\;\mathrm{node}\;\mathrm{availability}\;\mathrm{helper}\;\mathrm{vector}\;A,\;A=\left(a_{1},a_{2},\ldots ,a_{n}\right)$ with all available nodes for the path construction: A=(0,1,…,1).Repeat until n-th node is included in path π or more than n steps are evaluated:
$\mathrm{Find}\;\mathrm{the}\;\mathrm{index}\;\mathrm{of}\;\mathrm{subsequent}\;\mathrm{node}:\;next=\underset{i=1,\;\ldots ,n}{\arg\;\max}\left(w_{curr,i}^{\prime }\cdot p_{i}\cdot a_{i}\right);$$\mathrm{Include}\;\mathrm{node}\;v_{next}$ in path π;$\mathrm{Mark}\;\mathrm{the}\;\mathrm{included}\;\mathrm{node}\;\mathrm{as}\;\mathrm{unavailable}\;\mathrm{for}\;\mathrm{further}\;\mathrm{path}\;\mathrm{construction}:\;a_{next}=0$;Update current node index: curr=next.
$\mathrm{If}\;\mathrm{the}\;\mathrm{final}\;\mathrm{node}\;v_{n}$ was not included in path V, mark particle P as invalid.**Result:** path π corresponding to particle P (or invalid particle).

For example, consider the graphs presented in Figure 2 and Figure 3. The construction of the path for a random particle P=(0, 0.63, 0.3, 0.85, 0.94, 0.07, 0.73, 0.8, 0.08, 0.47, 1) begins with the assignment of a source node to the path $\pi =\left(v_{1}\right)$. As it is already included in the path, node $v_{1}$ is marked as unavailable (red color) for further evaluation. Then, the probabilities of all possible edges starting at the source node (according to the edge matrix $W^{\prime }$) are compared using element values of the particle P=(0, 0.63, 0.3, 0.85, 0.94, 0.07, 0.73, 0.8, 0.08, 0.47, 1). Node $v_{5}$ is appended to the path $\pi =\left(v_{1},\;v_{5}\;\right)$ because it has the highest probability (0.94 vs. 0.3) among all possible edges. Node $v_{5}$ is marked as unavailable for further path construction. Then, all possible edges starting from $v_{5}$ are evaluated in the same manner: P=(0, 0.63, 0.3, 0.85, 0.94, 0.07, 0.73, 0.8, 0.08, 0.47, 1). Node $v_{8}$ is added to the path $\pi =\left(v_{1},\;v_{5},\;v_{8}\right)$ because it has the highest probability (0.8). Finally, $v_{11}$ is added to the path as it has the highest probability compared to all other nodes reachable from node $v_{8}$: P=(0, 0.63, 0.3, 0.85, 0.94, 0.07, 0.73, 0.8, 0.08, 0.47, 1). The final path that corresponds to the given particle is $\pi =\left(v_{1},\;v_{5},\;v_{8},\;v_{11}\right)$.

### 3.2. AHP for Optimal Path Selection

**Figure 6:**
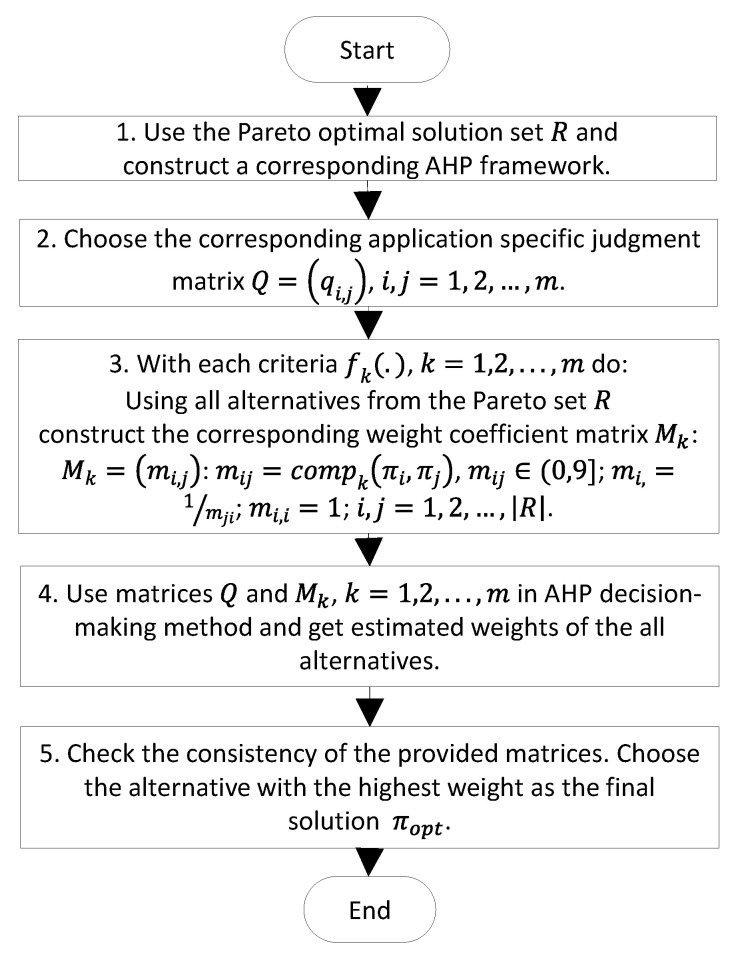
A flowchart of AHP for choosing the best path.

**Figure 7:**
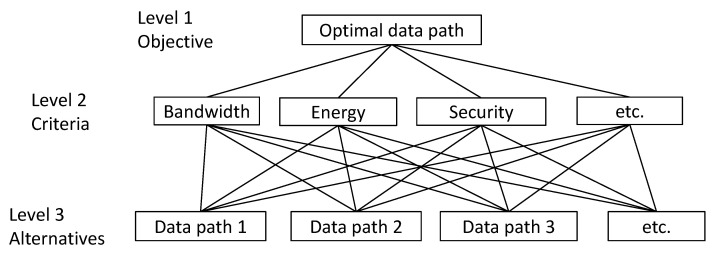
A hierarchical framework used for the AHP step.

AHP was used to choose the optimal path from the Pareto set. Figure 6 presents a generalized flowchart of AHP:

In step 1, a three-level AHP framework was constructed (Figure 7). The main objective of the process, that is, determining the best path from the source node to the destination node, comprised the first level. All objectives of the PSO optimization phase were formalized as criteria of AHP and became the second level. The weights of the criteria were calculated on the basis of a pairwise comparison usually conducted manually by experts in the application field. The final result of this step was the so-called judgment matrix (that is, matrix Q in Step 2), provided to the algorithm beforehand. All alternative paths from the Pareto set formed the third level of the AHP framework. In step 3, the weight coefficient matrices $M_{k}$, k=1, 2, …,m for each path from the Pareto set were formed by calculating their elements using a special comparison function $comp_{k}\left(\pi_{i},\pi_{j}\right)$. A comparison function uses the corresponding objective functions $f_{k}\left(\pi \right)$, calculates two values $f_{k}\left(\pi_{i}\right)$ and $f_{k}\left(\pi_{j}\right)$, compares them, and transforms the result to the value from the interval (0,9] required by AHP. These comparison functions depend heavily on the nature of the criteria and are defined specifically and differently for each criterion.

Then, AHP was started (step 4 in the flowchart) and one best path was selected as the final result (step 5 in the flowchart).

### 3.3. Objective Functions and Constraints

Different devices of IoT nodes have different performances, network bandwidths, security characteristics, etc. Therefore, the objective functions $f_{j}\left(\pi \right)$, j=1, 2,…,m and constraints $g_{k}\left(\pi \right)$ and $h_{l}\left(\pi \right)$ should be defined according to the situation in the real infrastructure. In the experiments presented in this paper, we used the following objective functions for the evaluation:

The total bandwidth used by the data traveling through the path π was calculated as the total weight of the graph edges, i.e., $f_{bw}\left(\pi \right)=w_{i_{1}i_{2}}+w_{i_{2}i_{3}}+\ldots$, where $\pi =\left(v_{i_{1}},v_{i_{2}},\;\ldots \right)$ is the data path under evaluation and $W=\left(w_{ij}\right)$ is the matrix of bandwidth usage. If the matrix W carries latency values, a similar equation is also applicable for a network-induced latency evaluation: $f_{lt}\left(\pi \right)=w_{i_{1}i_{2}}+w_{i_{2}i_{3}}+\ldots$.

Some objective functions could not be expressed by the total weight of the edges because their value depended on the nodes included in the path. For example, CPU and RAM use should be calculated using the expression $f_{CPU}\left(\pi \right)=w_{i_{1}}^{*}+w_{i_{2}}^{*}+\ldots$, where the weight vector $W^{*}=\left(w_{i}^{*}\right)$, i=1, 2,…,n represents CPU use in MIPS by the data transfer through the corresponding nodes. Similarly, $f_{RAM}\left(\pi \right)=w_{i_{1}}^{*}+w_{i_{2}}^{*}+\ldots$, where $W^{*}$ is the RAM-usage vector of the corresponding nodes expressed in MB.

The security objective function $f_{sec}\left(\pi \right)$ used in this paper was calculated using yet another expression. The security of the entire data transferred along the path π, that is, $f_{sec}\left(\pi \right)$, was defined by the lowest security of all nodes included in the path. We assigned security levels (expressed in security bits, according to the NIST publication [35]) to nodes on the basis of their ability to support the corresponding security protocols. In this case, $f_{sec}\left(\pi \right)=\underset{k}{\min}\left(512-w_{i_{k}}^{*}\right)$, where $W^{*}$ is the vector of the security values of the corresponding nodes. Expression 512-x was used because the PSO algorithm tried to minimize the objective function. Thus, better security should correspond to smaller values of the objective function.

Other application-specific objective functions may also be used, such as power requirements and energy consumption. The concrete definition may also vary according to the system characteristics important in a selected scenario. The proposed optimization method was not limited to any specific amount or nature of the objective functions, as long as they satisfied these two simple requirements:

- The result of the objective function is a positive real number.
- Better values of the criteria are expressed by smaller numbers (i.e., the PSO method searches for a minimum of the function).

The constraints were also specific application-dependent functions. For example, total memory consumption or CPU use could not exceed the physical capabilities of the corresponding node. If the application area required a specific level of security, then it should be expressed as a constraint, for example, $g_{sec}\left(\pi \right)\geq 128$, where $g_{sec}\left(\pi \right)$ is calculated in exactly the same manner as $f_{sec}\left(\pi \right)$ described above. During the PSO phase of optimization, particles that violate the constraints were assigned large fines and naturally eliminated from the optimization process.

## 4. Results and Discussion

In this section, we summarize the implementation results of the proposed method. The main objective was to evaluate the characteristics of the algorithm under different situations and to test the feasibility of using it in real-life scenarios.

The method proposed for determining the best path was implemented using MATLAB. As input, the implementation used graph data with several weight matrices and vectors used to calculate the values of multiple objective functions. All the concrete numbers used here were only for illustration purposes and did not have any specific meaning. To better understand the context, we called the first objective function the bandwidth evaluation function $f_{bw}$, the second objective function the latency function $f_{lt}$, and the third objective function the security function $f_{sec}$. All these objective functions were calculated as described in the previous section. The implemented version of the algorithm performed a multi-objective particle swarm optimization, found a Pareto optimal set of paths, automatically formed required comparison matrices used in AHP, and chose the best path using a provided judgment matrix.

**Figure 8:**
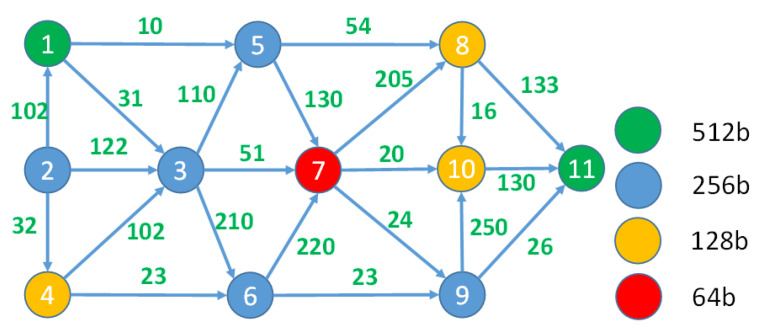
Latency (shown as the weights of the corresponding edges) and security (expressed in security bits and indicated by nodes of different colors) of the infrastructure represented as the graph.

To illustrate the proposed optimization method, we considered the example graph presented in Figure 2 (Graph A). Assume that the weights of the edges marked in blue represent the bandwidth requirements. In Figure 8, the same graph is supplemented with latency requirements marked in green near the edges and the security evaluation of the infrastructure elements, which are marked by different colors of the corresponding graph nodes.

Suppose our objective was to determine the best route from nodes $v_{1}$ to $v_{11}$ that ensured minimal total bandwidth usage and minimal total latency and also guaranteed maximal security. In this case, the three-dimensional objective function was $F\left(\pi \right)=\left(f_{bw}\left(\pi \right),f_{lt}\left(\pi_{i}\right),\;f_{sec}\left(\pi \right)\right)$. The PSO stage of the proposed method produced a Pareto set of nondominated solutions, as presented in Table 2.

If we used the one-dimensional PSO method to find the best paths using all three objective functions separately, then the results would be as follows: $\pi_{opt}=\pi_{3}$ if only bandwidth use was considered (in this case, the minimal bandwidth use would be 56); $\pi_{opt}=\pi_{2}$ if only latency was optimized (in this case, the best result should be 132); and $\pi_{opt}=\pi_{5}$ with 256 bits of total security if only security was optimized. As one can see, all optimal values of one-dimensional optimization cases are present in the Pareto set, complemented by some additional paths, which also may be chosen during the AHP step. The presence of the best values of one-dimensional optimization cases in the Pareto set indicates that the multi-objective optimization method works correctly and finds all the most important alternatives. During this step, a swarm of 20 particles was used and the number of iterations was 50.

The judgment matrix used during the AHP stage of optimization is:

(3) $$Q=\left(\begin{array}{ccc} 1 & 2 & 1/7\\ 1/2 & 1 & 1/3\\ 7 & 3 & 1 \end{array}\right).$$

This matrix means that minimal bandwidth consumption is more important than overall latency (2 vs. 1), but the security of the data path is much more important than both bandwidth and latency (7 and 3 vs. 1 accordingly). The results of the AHP evaluation of alternatives are summarized in Table 3.

The best path is $\pi_{opt}=\pi_{5}=\left(v_{1},\;v_{3},\;v_{6},v_{9},\;v_{11}\right)$, which also means that the best collection of values of the objective functions is (290, 101, 256).

In the second scenario, we used the graph that was evaluated by other authors [15,19,30]. We assumed that the standard edge weights used in one-dimensional optimization scenarios were bandwidth use.

**Figure 9:**
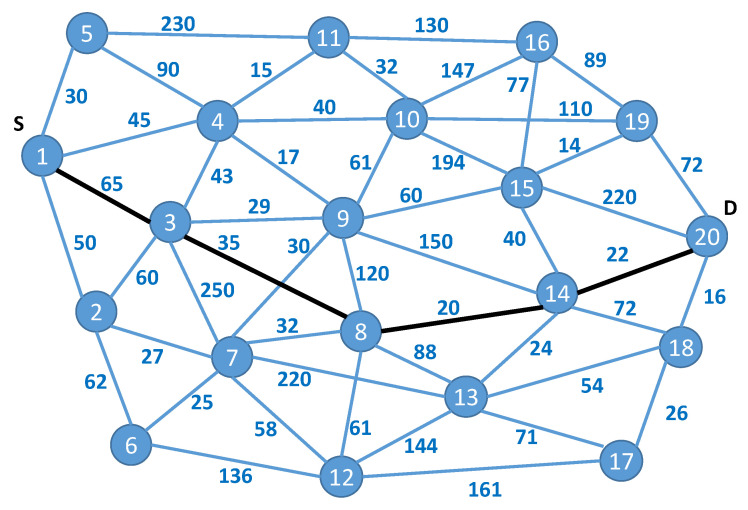
The graph used in the second scenario with bandwidth requirements near the corresponding edges (graph B).

In Figure 9, the optimal path, considering only one objective function, found by the algorithm proposed by the authors of [15] is shown by the bold lines. The total weight of this path is 142. Moreover, other algorithms have found only suboptimal paths: Munetomo’s [32] algorithm found a path with a total weight of 187 and Inagaki’s [36] algorithm found one with a weight of 234.

**Figure 10:**
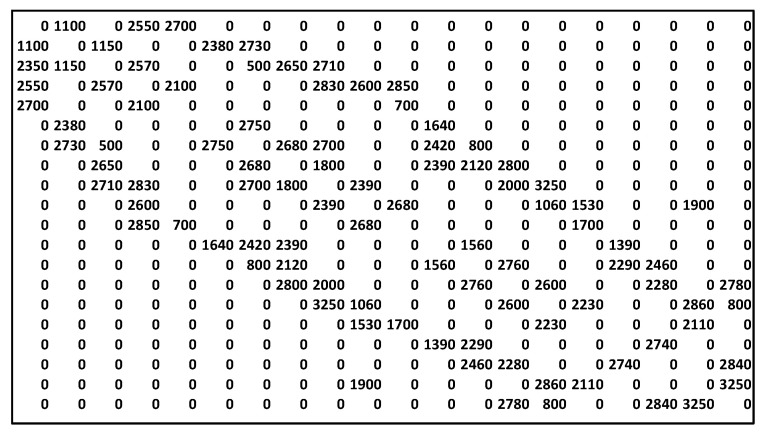
The latency values for the edges of graph B.

To use multi-objective optimization, we added a second set of weights (i.e., latency) to the edges and defined the security levels of the nodes. Figure 10 presents the corresponding weight matrix. For the AHP stage, we used the following judgment matrix:

(4) $$Q=\left(\begin{array}{cc} 1 & 5\\ 1/5 & 1 \end{array}\right).$$

**Figure 11:**
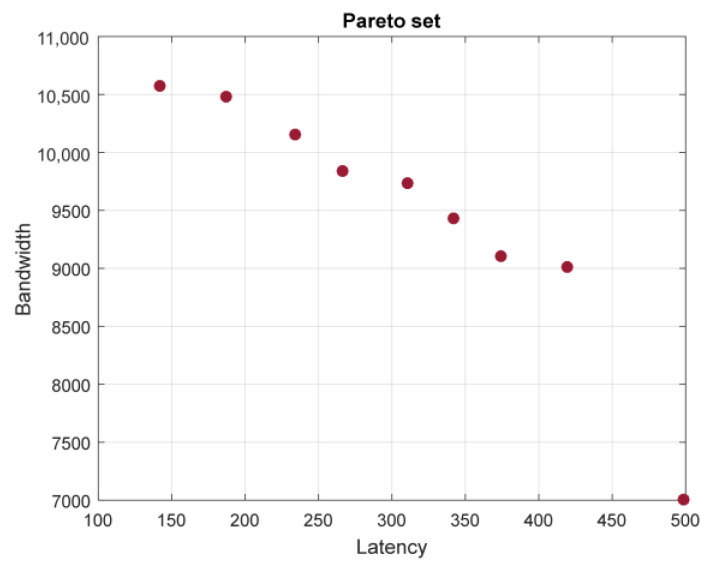
The Pareto front of the two-dimensional optimization of graph B.

Figure 11 presents a graph of the Pareto front while optimizing using only bandwidth and latency objective functions.

The complete results are summarized in Table 4, with the AHP evaluation scores added as the fifth column. We used a swarm of 40 particles and 50 iterations for the PSO part of the optimization.

The best alternative was $\pi_{7}$. One can easily view all the optimal and suboptimal paths (considering only bandwidth objective function) discussed above among the members of the Pareto set (the optimal path while using one-dimensional optimization according to latency was 7010).

To show the influence of the judgment matrix on the final result, we used all three objective functions and two different judgment matrices. Matrix $Q_{1}$ prioritizes security:

(5) $$Q_{1}=\left(\begin{array}{ccc} 1 & 2 & 1/7\\ 1/2 & 1 & 1/3\\ 7 & 3 & 1 \end{array}\right).$$

The second judgment matrix, that is, $Q_{2}$, prioritizes bandwidth over all other objectives:

(6) $$Q_{2}=\left(\begin{array}{ccc} 1 & 5 & 2\\ 1/5 & 1 & 1/3\\ 1/2 & 3 & 1 \end{array}\right).$$

**Figure 12:**
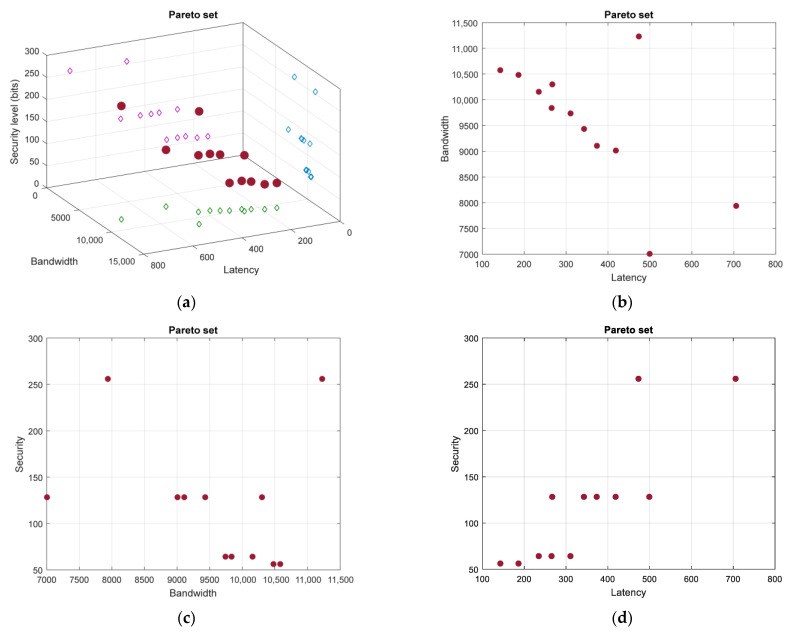
The Pareto set for graph B (three objective functions are used): (**a**) three-dimensional view of the Pareto set; (**b**) bandwidth–latency projection of the Pareto set; (**c**) security–bandwidth projection of the Pareto set; and (**d**) security–latency projection of the Pareto set.

The description of graph B was complemented by the node security vector $W^{*}=\left(512,\;256,\;128,\;128,\;256,\;128,\;256,\;56,\;128,\;256,\;256,\;128,\;64,\;64,\;256,\;128,\;64,\;128,\;256,\;512\right)$. During the PSO stage of optimization, we used a particle swarm of 40 particles and 50 iterations. Figure 12 presents the Pareto set of solutions.

If the judgment matrix $Q_{1}$ is used for the AHP step, then the best path is $\pi_{1}=\left(v_{1},\;v_{5},\;v_{11},v_{10},\;v_{15},v_{20}\right)$, with the following results of the objective functions: $f_{bw}\left(\pi_{1}\right)=706$, $f_{lt}\left(\pi_{1}\right)=7940$, and $f_{sec}\left(\pi_{1}\right)=256$. However, if the judgment matrix $J_{2}$ is used, then the best path is $\pi_{2}=\left(v_{1},\;v_{3},\;v_{8},v_{14},v_{20}\right)$, with the corresponding objective functions having the following scores: (142, 10, 580, 56).

**Figure 13:**
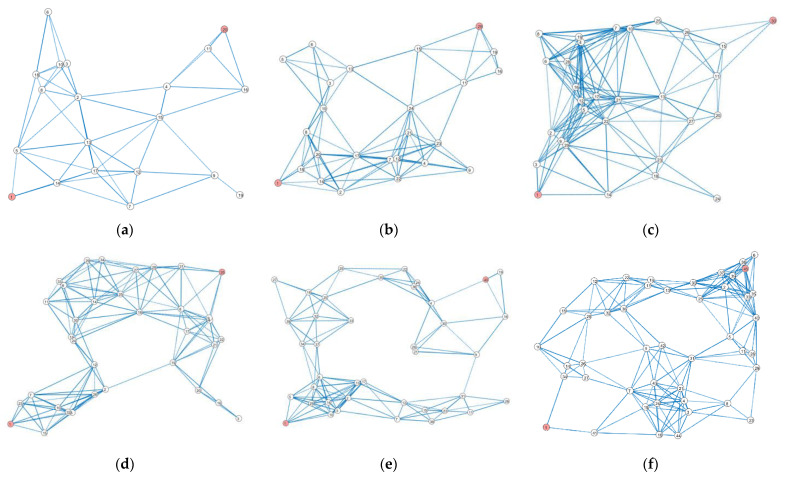
The randomly generated graphs used for evaluating the proposed method: (**a**) Graph20, with 20 nodes and 62 edges; (**b**) Graph25, with 25 nodes and 102 edges; (**c**) Graph30, with 30 nodes and 167 edges; (**d**) Graph35, with 35 nodes and 156 edges; (**e**) Graph40, with 40 nodes and 152 edges; and (**f**) Graph45, with 45 nodes and 206 edges.

To test the proposed algorithm with graphs of different sizes, we generated some random graphs with random weight values assigned to the edges (representing bandwidth and latency) and nodes. Graphs with different numbers of nodes (from 20 to 45) are presented in Figure 13. For example, Figure 13a presents Graph20, with 20 nodes, and Figure 13b presents Graph25, with 25 nodes. Table 5 summarizes the results when the proposed method is applied to all these graphs. The judgment matrix used during the evaluation was $Q_{2}$ from Equation (3).

The experimental evaluation shows that the proposed method effectively finds the Pareto front in cases with graphs containing up to 45 nodes. If the graph size increases, then the PSO stage of the algorithm is not as effective because, in some cases, the method behaves in an unstable manner, i.e., in some cases, it does not include optimal paths in the Pareto set.

## 5. Conclusions

In this paper, we proposed a novel approach for finding the optimal data path in a heterogeneous IoT infrastructure. The proposed two-stage method used multi-objective particle swarm optimization to find a Pareto optimal set of alternative data paths, and then an analytical hierarchy process was applied to select the best alternative. The alternatives were evaluated using judgment matrices created once experts evaluated the optimization criteria used during the process. This approach had a double-fold effect: (1) it allowed us to compare different criteria, which is always challenging because the criteria may differ in that they may be qualitative, quantitative, use different units of measurement, etc.; and (2) in different application areas, the objective functions may differ in terms of importance. In such an instance, a different judgment matrix, prepared beforehand by experts in the corresponding application area, is sufficient to modify the method to be used in different scenarios. Moreover, the proposed method not only provided the whole set of alternative solutions, but also evaluated each of them. It allowed us to choose the second- or third-best alternative if the first was not suitable for some reason.

The proposed method worked with a wide range of objective functions, which can be easily expanded. In the examples presented in this article, we used two methods to evaluate objective functions. One can easily combine both approaches or even define more complex or even dynamic objective functions. The proposed approach was transparent as to the nature of the objective function, as long as two simple requirements were met: the result of the objective function was a positive, real number and better values of the criteria were expressed by smaller numbers (i.e., the PSO method searches for a minimum of the function).

The main advantages of the proposed method were its simplicity and the fact that it can be adapted to limited available resources, because both algorithms used during the two stages were well-suited to the constrained nature of fog devices. If the calculation characteristics of the fog node are limited, then the PSO algorithm can be used with fewer particles and/or iterations. Even in such cases, some suboptimal solutions would be found and provide “good enough” results. In addition, the second stage of the proposed method (AHP) was a simple deterministic method, which always chose the best alternative from the given set.

If the complexity of the graph representing the IoT infrastructure did not exceed 40 nodes and 120 edges, then the proposed algorithm produced a Pareto set of alternatives that included all alternatives with all optimal paths while considering each objective separately. If the complexity of the graph increased, the effectiveness of the PSO part of the algorithm was not sufficient. This limitation was not critical, considering the nature of the application of the proposed method, i.e., the IoT infrastructure. The graphs generated from real IoT devices will not exceed a few dozens of nodes and edges.

It was difficult to compare the proposed method with other similar optimization methods because few of them produced the full Pareto set. Usually, some kinds of combining functions are used during the search for an optimal solution. We tried to assess the correctness of the final Pareto set by applying the single-objective path optimization methods. The experimental results show that all the best paths found using all objective functions individually are also present in the set of Pareto Frontier. This shows that the proposed method successfully finds alternatives that are known to be nondominated beforehand.

Several interesting aspects of the proposed method could be explored in the future. It would be interesting to use it in a real IoT infrastructure and evaluate the number of resources saved or the level to which the QoS is improved. Furthermore, the construction of objective functions could be investigated and adapted to real measurements of real hardware.

We believe that the results of this work will be useful in future research in the area of IoT fog computing, data path optimization, and service orchestration, and will allow us to develop more efficient IoT systems.

## Figures and Tables

**Table 1:** The key notations used in this paper.

Notation	Description
G=(V,E)	$\mathrm{Graph}\;\mathrm{representing}\;\mathrm{the}\;\mathrm{IoT}\;\mathrm{infrastructure}.\;V=\left\{v_{i}\right\}$ is a set of nodes and E∈V×V is a set of edges.
$\pi_{i}=\left(v_{i1},v_{i2},\;\ldots ,v_{ik}\right)$	The i-th path for data transfer from the source to final node.
W	Edge weight matrix representing capabilities of corresponding IoT infrastructure links (bandwidth, latency, etc.).
n	Total number of nodes; also the dimensionality of the objective function definition area.
$n_{p}$	Total number of particles.
$P_{i}=\left(p_{1},p_{2},\ldots ,p_{n}\right)$, $\;p_{j}\in R$$,\;p_{j}\geq 0$, j=1, 2, …,n	Position of the i-th particle in the n-dimensional definition area.
D, $\pi_{i}=D\left(P_{i}\right)$	Path decoding function used to decode a particle into a path.
m	Total number of evaluation criteria; also the number of objective functions.
$f_{j}\left(\pi \right)$ , j=1, 2,…,m	The j-th evaluation criterion; also the objective function.
$F\left(\pi_{i}\right)=\left(f_{1}\left(\pi_{i}\right),f_{2}\left(\pi_{i}\right),\;\ldots ,f_{m}\left(\pi_{i}\right)\right)$	Score vector of the i-th path or i-th particle.
$U_{i}$ $,\;U_{i}\in R^{n}$	Velocity of the i-th particle.
$pBest_{i}$	The best score of the i-th particle.
$pBPos_{i}$	The best position of the i-th particle.
gBest	The best global score of all the particles.
gBPos	Position of the particle with the best global score.
S	Set of particles (swarm).
R	Set of Pareto optimal solutions.
$\pi_{opt}$ $,\;P_{opt}$	Best path and the particle with the best score.
Q	Judgment matrix with the results of the pairwise criteria comparison used in the AHP.
$comp_{k}\left(\pi_{i},\pi_{j}\right)$, k=1, 2,…,m	Function of the pairwise comparison of two paths using the k-th criterion.
$M_{k}$ , k=1, 2, …,m	$\mathrm{Weight}\;\mathrm{coefficient}\;\mathrm{matrix}\;\mathrm{with}\;\mathrm{pairwise}\;\mathrm{comparisons}\;\mathrm{using}\;\mathrm{comparison}\;\mathrm{function}\;comp_{k}$ on all paths from the Pareto set *R.*

**Table 2:** The Pareto set of the alternative paths for Graph A.

Alternative Path	Total Bandwidth, $f_{bw}\left(\pi \right)$	Total Latency, $f_{lt}\left(\pi \right)$	Security, $f_{sec}\left(\pi \right)$
$\pi_{1}=\left(v_{1},\;v_{5},\;v_{8},\;v_{11}\right)$	77	197	128
$\pi_{2}=\left(v_{1},\;v_{3},\;v_{7},v_{9},\;v_{11}\right)$	132	132	128
$\pi_{3}=\left(v_{1},\;v_{5},\;v_{7},v_{8},\;v_{11}\right)$	56	478	128
$\pi_{4}=\left(v_{1},\;v_{5},\;v_{7},v_{10},\;v_{11}\right)$	56	290	128
$\pi_{5}=\left(v_{1},\;v_{3},\;v_{6},v_{9},\;v_{11}\right)$	101	290	256
$\pi_{6}=\left(v_{1},\;v_{5},\;v_{7},v_{9},\;v_{11}\right)$	73	190	128

**Table 3:** The AHP scores of the alternative paths.

Alternative	$\pi_{1}$	$\pi_{2}$	$\pi_{3}$	$\pi_{4}$	$\pi_{5}$	$\pi_{6}$
AHP score	0.144	0.113	0.088	0.098	0.450	0.106

**Table 4:** The Pareto set of the alternative paths for graph A.

Alternative Path	$\mathrm{Total}\;\mathrm{Bandwidth},\;f_{bw}\left(\pi \right)$	Total Latency, $f_{lt}\left(\pi \right)$	AHP Score
$\pi_{1}=\left(v_{1},\;v_{2},\;v_{3},v_{8},\;v_{14},v_{20}\right)$	187	10,480	0.20
$\pi_{2}=\left(v_{1},\;v_{4},\;v_{9},\;v_{14},v_{20}\right)$	234	10,160	0.13
$\pi_{3}=\left(v_{1},\;v_{4},\;v_{9},\;v_{15},v_{20}\right)$	342	9430	0.05
$\pi_{4}=\left(v_{1},\;v_{3},\;v_{9},\;v_{14},v_{20}\right)$	266	9840	0.09
$\pi_{5}=\left(v_{1},\;v_{3},\;v_{9},\;v_{15},v_{20}\right)$	374	9110	0.05
$\pi_{6}=\left(v_{1},\;v_{4},\;v_{10},\;v_{15},v_{20}\right)$	499	7010	0.08
$\pi_{7}=\left(v_{1},\;v_{3},\;v_{8},\;v_{14},v_{20}\right)$	142	10,580	0.30
$\pi_{8}=\left(v_{1},\;v_{2},\;v_{3},v_{9},\;v_{15},v_{20}\right)$	419	10,580	0.05
$\pi_{9}=\left(v_{1},\;v_{2},\;v_{3},v_{9},\;v_{14},v_{20}\right)$	311	9740	0.05

**Table 5:** The best paths found by the method proposed for the graphs presented in Figure 13.

	Nodes	Edges	Best Path	$f_{bw}\left(\pi \right)$	$f_{lt}\left(\pi \right)$	$f_{sec}\left(\pi \right)$	Particles	Iterations
Graph20	20	62	$\pi =\left(v_{1},\;v_{5},\;v_{2},v_{4},v_{20}\right)$	135	908	56	40	50
Graph25	25	102	$\pi =\left(v_{1},\;v_{2},\;v_{7},v_{24},\;v_{11},v_{25}\right)$	196	898	128	40	55
Graph30	30	167	$\pi =\left(v_{1},\;v_{2},\;v_{21},v_{27},\;v_{11},v_{30}\right)$	153	1150	56	50	60
Graph35	35	156	$\pi =\left(v_{1},\;v_{10},\;v_{12},v_{24},\;v_{18},v_{31},v_{35}\right)$	207	1361	128	50	70
Graph40	40	152	$\pi =\left(v_{1},\;v_{8},\;v_{37},v_{32},\;v_{20},v_{35},v_{4},v_{40}\right)$	275	1560	56	60	90
Graph45	45	206	$\pi =\left(v_{1},\;v_{41},\;v_{7},v_{9},\;v_{31},v_{29},v_{40},v_{45}\right)$	215	2170	448	65	150

## Data Availability

Not applicable.
